# Distinct Cytokine Profiles in Lupus Low Disease Activity State Subgroups Identify Patients at Risk for Disease Flare

**DOI:** 10.3390/ijms27093913

**Published:** 2026-04-28

**Authors:** Warot Piriyasanguanpong, Boonjing Siripaitoon, Siriporn Juthong, Parichat Uea-Areewongsa, Porntip Intapiboon

**Affiliations:** Allergy and Rheumatology Unit, Faculty of Medicine, Prince of Songkla University, Songkhla 90110, Thailand; warotpiri@gmail.com (W.P.); boonjing39@yahoo.com (B.S.); jusiripo@medicine.psu.ac.th (S.J.); uparicha@medicine.psu.ac.th (P.U.-A.)

**Keywords:** systemic lupus erythematosus, SLE, lupus low disease activity state, LLDAS, cytokine, interleukin-6

## Abstract

We compared the cytokine profiles between two lupus low disease activity state (LLDAS) subgroups—clinically active (CA) and serologically active clinically quiescent (SACQ)—and identified predictors of disease flare. Fifty patients with systemic lupus erythematosus (25 CA, 25 SACQ) who maintained LLDAS for ≥6 months were enrolled and followed for 6 months. Cytokine modules were identified using weighted gene co-expression network analysis, and correlations with clinical traits were assessed. Predictors of flare were assessed using Cox regression. Three cytokine modules were identified. The brown (MCP-1 and IL-8) and turquoise (IFN-α, IFN-γ, IL-17A, IL-10, IL-12p70, IL-18, IL-23A, and IL-33) modules correlated with mucocutaneous and physician global assessment, respectively. These modules showed a positive, but not significant, correlation with CA. The comparison analysis revealed that IL-6 and IL-8 were higher in CA than in SACQ. Nine patients (18%) flared, six of whom belonged to the CA group. Flares were associated with a lower sustained LLDAS rate (77.8% vs. 34.1%) and higher levels of IL-1β, IL-6, and IL-33. In multivariable analysis, non-sustained LLDAS (HR 8.73) and IL-6 ≥ 45.1 pg/mL (HR 10.4) independently predicted a flare. Our study demonstrated that cytokine elevation persists despite LLDAS. Non-sustained LLDAS and elevated IL-6 predict a flare, suggesting that IL-6 may enhance the flare prediction biomarker.

## 1. Introduction

Achieving disease remission with no clinical disease activity, irrespective of serology—as defined by The Definitions Of Remission In Systemic Lupus Erythematosus (DORIS) [1]—has been stated as the treatment goal for systemic lupus erythematosus (SLE) in the current international recommendations [2,3,4]. Notably, 33.7–54.0% of patients with SLE achieve this target [5,6]. However, increased use of immunosuppressive drugs is associated with a higher percentage of infectious complications. Lupus Low Disease Activity State (LLDAS) is an alternative targeted treatment goal proposed by Franklyn et al. [7]. In addition, a validation study demonstrated that prolonged LLDAS duration is associated with a lower risk of disease flare-ups, reduced organ damage accrual, and improved quality of life compared with persistent disease activity [8]. LLDAS encompasses a range of clinical and serological manifestations, including low disease activity, defined by the Systemic Lupus Erythematosus Disease Activity Index 2000 (SLEDAI-2K) of ≤4, with no activity in major organ systems; Safety of Estrogens in Lupus Erythematosus National Assessment (SELENA)-SLEDAI physician global assessment (PGA) (scale 0–3) ≤1; and use of glucocorticosteroids (GCs) equivalent to prednisolone doses of ≤7.5 mg/day and stable immunosuppressive drugs and/or biologic agents. However, some patients achieving LLDAS were in a state with absent clinical symptoms but with serological markers indicating disease activity, defined as Serologically Active Clinically Quiescent (SACQ), which differs from DORIS remission in that the upper limit of prednisolone is ≤7.5 mg/day.

Studies on serum biomarker studies, beyond anti-DNA and complement studies, such as those on interferon (IFN)-α and IFN-γ; interleukin (IL)-6, IL-8, and IL-18; and monocyte chemotactic protein (MCP)-1 have focused on comparing disease activity, active, inactive, SACQ, LLDAS, and DORIS remission [9,10,11,12]. However, studies comparing LLDAS subgroups and flare risk in these states are scarce. Therefore, this study aimed to evaluate cytokine levels in LLDAS and to compare them between the Clinically Active (CA) and SACQ states. This study addresses the knowledge gap by comparing cytokine levels in patients with SLE across LLDAS subgroups and by identifying clinical or biomarkers that predict disease flare-ups.

## 2. Results

### 2.1. Participants’ Characteristics

The study flow diagram is illustrated in Figure S1. A total of 50 patients with SLE who met the eligibility criteria were consecutively enrolled, with 25 patients per group. Most patients with SLE were women (92%), with a mean age of 30.5 ± 10.7 years. The median disease duration was 5.4 years (interquartile range [IQR] 2.4, 15.3) in the CA group and 8.6 years (6.2, 15.8) in the SACQ group.

Mucocutaneous and musculoskeletal conditions were the most frequent clinical manifestations of CA. The other baseline characteristics of both groups were similar, except for three key differences: patients in the SACQ group had a longer LLDAS duration (years) [4.6 (3.1, 7.3) vs. 2.5 (0.6, 3.4), *p* = 0.02], a lower SELENA-PGA [0.5 (0, 1) vs. 1 (0.5, 1), *p* = 0.015], and a higher number of patients using azathioprine (AZA) [16 (64.0%) vs. 7 (28.0%), *p* = 0.02; Table 1].

### 2.2. Higher IL-6 and IL-8 Levels in Patients with Clinically Active than in Those with Serologically Active Clinically Quiescent

**Figure 1:**
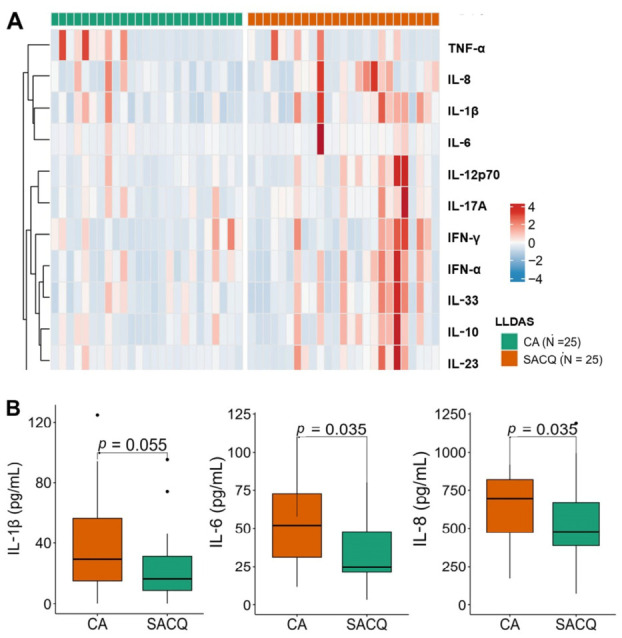
Diagram comparing cytokine levels between the CA and SACQ groups: (**A**) showed the cytokine levels between the groups of 50 LLDAS patients; (**B**) showed the significant cytokine levels between the CA and SACQ groups. IFN, interferon; IL, interleukin; CA, Clinically Active; LLDAS, Lupus Low Disease Activity State; SACQ, Serologically Active Clinically Quiescent; TNF, tumor necrosis factor.

Cytokine analysis showed that most values exceeded the normal range in the healthy individuals, with IL-6, IL-8, IL-18, IL-33, and MCP-1 reaching 3–5 times above the upper normal range. The heatmap (Figure 1A) illustrates notable elevated cytokines—particularly IL-8, IL-1β, IL-12p70, IFN-γ, IFN-α, IL-33, IL-10, and IL-23—in the CA group. Among these, IL-6 and IL-8 were significantly higher in CA than in SACQ [59.9 (32.9–91.5) pg/mL vs. 27.3 (21.3–58.6) pg/mL, *p* = 0.035; and 2179.7 (818.1–3823.4) pg/mL vs. 1190.5 (476.6–1610.8) pg/mL, *p* = 0.049, respectively; (Figure 1B, Table S1)]. IL-6 and IL-8 were subsequently selected as candidate biomarkers for flare-risk modeling. 

### 2.3. Weighted Gene Co-Expression Network Analysis Identified Three Cytokine Modules and Module–Trait Correlation; Three Cytokines Correlated with Disease Activity Status

**Figure 2:**
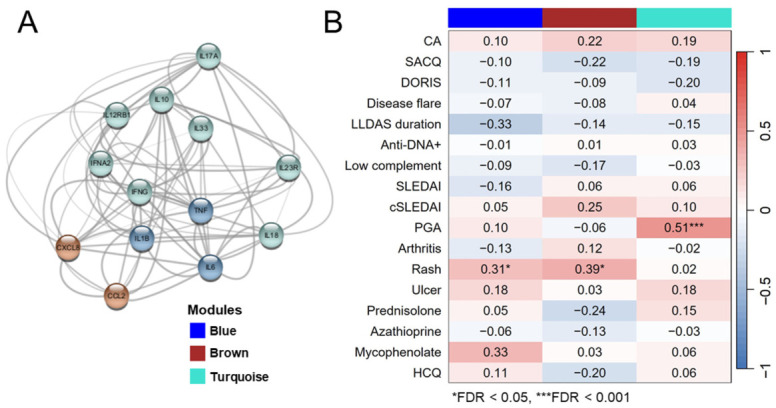
Cytokine modules from weighted gene co-expression network analysis and module–trait correlation: (**A**) showed the cytokines interaction; (**B**) showed the module–trait correlations. CA, clinically active; DORIS, Definitions of Remission in Systemic Lupus Erythematosus; FDR, False Discovery Rate; HCQ, hydroxychloroquine; LLDAS, Lupus Low Disease Activity State; PGA, Physician’s Global Assessment; SACQ, Serologically Active Clinically Quiescent; SLEDAI, Systemic Lupus Erythematosus Disease Activity.

**Figure 3:**
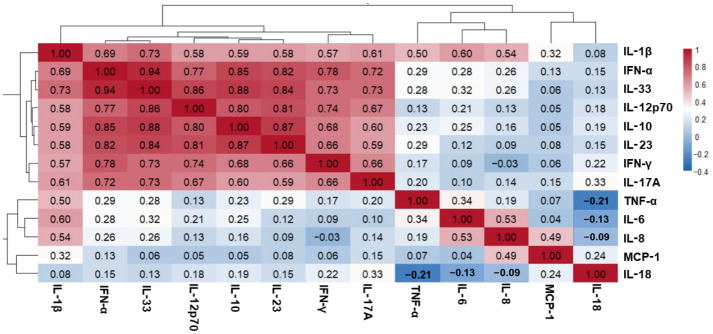
Spearman’s rank correlation coefficient shows the correlation between cytokine–cytokine interactions. IFN, interferon; IL, interleukin; MCP, monocyte chemotactic protein; TNF, tumor necrosis factor.

To explore the relationship between cytokine clusters and clinical variables and to identify potential biomarkers predicting disease flare-up, a co-expression network was constructed using 13 cytokines and a soft-thresholding power of 1. Three co-expression modules were identified (Figure 2A): blue: IL-1β, IL-6, and tumor necrosis factor (TNF)-α; brown: MCP-1 and IL-8; and turquoise: IFN-α, IFN-γ, IL-17A, IL-10, IL-12p70, IL-18, IL-23A, and IL-33. Module–trait correlation analysis revealed that the brown and turquoise modules were positively correlated with the CA group (*r* = 0.22 and 0.19, respectively) and inversely correlated with SACQ and DORIS. However, these correlations were not statistically significant. The blue module showed a negative correlation with sustained LLDAS (*r* = −0.33, FDR = 0.05) but a positive correlation with mucocutaneous involvement (*r* = 0.31, FDR = 0.04). Additionally, mucocutaneous manifestations positively correlated with the brown module (*r* = 0.39, FDR = 0.01), whereas the turquoise module showed a strong correlation with PGA (*r* = 0.51, FDR < 0.001) (Figure 2B). At the single-cytokine level, the PS score revealed that IL-10 and IL-8 were positively correlated with CA (*r* = 0.29, FDR = 0.045; *r* = 0.27, FDR = 0.055, respectively), whereas IL-1β was negatively correlated with SACQ (*r* = −0.35, FDR = 0.012). However, none of these cytokines were significantly associated with disease flare. IL-1β had a negative correlation with sustained LLDAS (*r* = −0.32, FDR = 0.023), whereas IL-18 was negatively correlated with anti-dsDNA (*r* = −0.39, FDR = 0.005). Interestingly, various cytokines, including IFN-α, IFN-γ, IL-17A, IL-10, IL12p70, IL-23, and IL-33, were correlated with PGA (*r* = 0.32–0.69; FDR < 0.024 to <0.001). The protein score (PS) summary with the interesting clinical trait is presented in Supplementary Table S2. The correlation pattern from WGGNA concords with the Spearman’s correlation from conventional analysis, which revealed three clusters of cytokines with moderate-to-strong intercorrelations (*r* > 0.6, *p* < 0.001) (Figure 3 and Figure S2).

### 2.4. The Impact of Immunosuppressive Drugs on Cytokine Levels: Correlation Analysis and Analysis of Covariance

We performed a correlation analysis of cytokine–immunosuppressive drug use using PS scores derived from WGCNA (Table S2). GCs use demonstrated negative correlations with MCP-1 and IL-8 (*PS* = −0.25 and −0.17, respectively), although these were not statistically significant. AZA and hydroxychloroquine (HCQ) also showed negative correlations with IL-8 (*PS* = −0.26 for both), with borderline significance (FDR = 0.068 and 0.071, respectively). In contrast, mycophenolate mofetil (MMF) demonstrated significant positive correlations with IL-6 and IL-1β (*PS* = 0.40, FDR = 0.004; and *PS* = 0.31, FDR = 0.028, respectively). To evaluate potential confounding effects of immunosuppressive therapy, we performed an analysis of covariance (ANCOVA), adjusting for drug use and their interactions with LLDAS status (Table S3). GCs showed no significant effect on MCP-1 and no interaction with the subgroup. In contrast, GCs significantly modified IL-8 levels, with significant interactions observed at both ≥5 mg/day and ≥7.5 mg/day thresholds (interaction *p* = 0.004). Although IL-8 levels were higher in the CA group, this difference was attenuated in patients receiving higher-dose prednisolone. Despite a higher proportion of AZA use in the SACQ group, IL-6 and IL-8 remained significantly higher in the CA group after adjustment (IL-6: *p* = 0.026; IL-8: *p* = 0.037). Therefore, AZA use was not independently associated with either cytokine. Similarly, after adjusting for MMF use, IL-6 remained significantly higher in the CA group (*p* = 0.027), whereas IL-1β showed a non-significant trend (*p* = 0.080). MMF use was not independently associated with cytokine levels, and no significant interaction with subgroup was observed.

### 2.5. Higher 6-Month Disease-Flare Rate in Patients with Clinically Active than in Those with Serologically Active Clinically Quiescent LLDAS

All participants were prospectively followed up, and SLEDAI-2K scores were evaluated for at least 6 months after completing the cytokine measurement. Nine patients experienced disease flare-ups. Six patients (66.7%) were from the CA group. Of these, three had lupus nephritis, five had mucocutaneous or articular flare-ups, and one had gastrointestinal vasculitis. Three patients met the definition of severe flare according to SELENA-SFI, and one was managed by increasing GCs doses and adjusting immunosuppressive therapy. The median SLEDAI-2K score was 8 (IQR 6–16). Six patients had low complement levels, and all had IL-6 levels above the normal range (Table S4).

### 2.6. Identifying the Clinical Factors and Cytokine Levels Predicting Short-Term Disease Flare-Ups

We compared the clinical characteristics of patients with disease flare-ups and those in a clinically stable state. The median duration (year) of LLDAS in the flare-up group was significantly shorter than that in the stable state group [IQR 1.9 (0.8, 3) vs. 3.4 (IQR 2, 7.3) years, *p* = 0.04]. Additionally, the mean LLDAS-to-total follow-up duration ratio was only 0.3 (standard deviation [SD] = 0.3), compared with 0.6 (SD = 0.3, *p* = 0.02), corresponding to a higher proportion of patients with non-sustained LLDAS (77.8% vs. 34.1%, *p* = 0.03). We then compared cytokine levels between the two groups and found no differences; however, we observed higher median levels of IL-1β, IL-6, and IL-33 in the disease flare-up group, whereas MCP-1, IL-8, and IL-10 were notably higher in the inactive group (Table 2).

### 2.7. Higher IL-6 and Non-Sustained LLDAS Are Prognostic Factors of Short-Term Disease Flare-Ups in Patients with SLE with LLDAS

**Figure 4:**
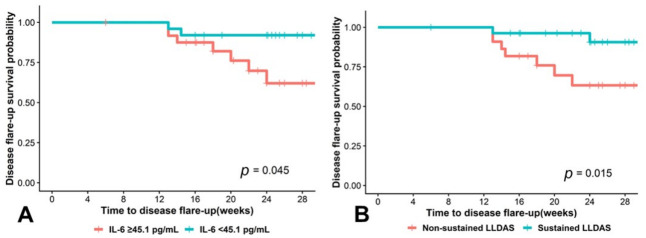
The Kaplan–Meier graph demonstrates the survival probability of disease flare within 6 months in patients with systemic lupus erythematosus: (**A**) IL-6 ≥ 45.1 pg/mL and (**B**) non-sustained LLDAS. IL, interleukin; LLDAS, lupus low disease activity state.

We determined cytokine cutoffs to distinguish between disease flares and the clinically stable state (Table S5). After identifying appropriate cytokine cutoff values, univariable and multivariable analyses were performed using these values and statistically significant clinical factors to identify predictors of disease flare-ups within 6 months. In the best-fitting model, IL-8 and MCP-1 were excluded from the univariable and multivariable analyses due to a sensitivity of 1 and very low specificity, which makes them unsuitable for calculation. The univariable analysis identified factors with a *p*-value < 0.1, including non-sustained LLDAS, IFN-γ ≥ 97.3 pg/mL, IL-6 ≥ 45.1 pg/mL, IL-10 ≥ 130.0 pg/mL, and IL-23 ≥ 81.7 pg/mL. These factors were then included in the multivariable analysis. The final multivariable model identified two significant predictors of disease flare-ups within 6 months: non-sustained LLDAS and IL-6 levels ≥ 45.1 pg/mL (Table 3). However, the small number of patients with disease flare resulted in higher HRs with wide 95% confidence intervals; therefore, definitive conclusions regarding these predictive factors cannot be drawn. The Kaplan–Meier graph demonstrated the disease-flare survival probability within 6 months in patients with SLE with IL-6 ≥ 45.1 pg/mL (Figure 4A) [survival 62.1% vs. 90.8%, (95% confidence interval [CI] = 0.426–0.905), *p* = 0.015)] and the non-sustained LLDAS [survival 61.1% vs. 91.1%, (95% CI = 0.418–0.895), *p* = 0.045)] (Figure 4B).

## 3. Discussion

This study delineated distinct immunological profiles among patients with SLE across LLDAS subgroups, with higher levels of inflammatory cytokines observed in clinically active disease than in SACQ. We identified IL-6—a previously reported biomarker—as a candidate biomarker that correlated with disease flare-ups, in conjunction with a previously reported clinical predictor—LLDAS sustainment. In addition, we observed that patients in the CA group experienced flares more frequently than those in the SACQ group.

The study results revealed that patients with SLE in the LLDAS group exhibited predominantly elevated cytokine levels, including IFN, IL-6, IL-8, IL-18, IL-33, and MCP-1, above the upper normal limit of a similar healthy population, which suggests that patients with SLE have ongoing immune system abnormalities, even in a state of low disease activity or SACQ, particularly concerning IL-6 and IL-8 levels, which are higher in clinically active patients. These comparative analysis results are consistent with the WGCNA, which revealed a positive correlation module of various cytokines in the clinically active. This finding is consistent with the latest transcriptomic analysis by Parodis et al. [13], which revealed that patients with LLDAS or low SLEDAI-2K scores showed a positive association between immunological functions and reactome pathways compared to those in DORIS remission. However, that study did not stratify LLDAS as SACQ or CA, as our study did. In addition to disease activity subgroups, we demonstrated correlations between PGA scores and several cytokines (IFN-α, IFN-γ, IL-10, IL-12p70, IL-23A, and IL-33). These cytokines represent upstream immune mediators that modulate interferon production through multiple intracellular signaling pathways, particularly the JAK–STAT pathway [14]. Thus, PGA may serve as an important tool for detecting subtle disease activity in patients with LLDAS, as it reflects not only clinical disease activity [15] but also underlying immunological activity.

Previous studies have shown that IL-6 levels are elevated in patients with SLE compared with healthy controls and correlate with disease activity indices, such as SLEDAI, as well as with serum IFN-α levels [16,17,18,19]. However, these studies were conducted in patients, not limited to those with LLDAS. In contrast, our study demonstrates a distinct elevation of IL-6 in patients with clinically active disease despite maintaining LLDAS. These findings suggest that IL-6 may retain immunological aberration and clinical relevance even during states of low disease activity. In addition, IL-6 may serve as a predictive biomarker for disease flare-ups, as elevated IL-6 preceded disease activity, as demonstrated in our study. Apart from biomarkers, an essential clinical factor was a non-sustained LLDAS, which was significantly associated with disease flare-ups. This finding aligns with previous studies in both inception and non-inception cohorts, which reported that achieving an LLDAS sustainment of at least 50% protects against disease relapse [20,21]. Integration of IL-6 assessment with clinical parameters and LLDAS durability may enhance the precision of disease activity assessment in patients with SLE beyond conventional approaches; however, this interpretation should be taken with caution due to the small sample size.

The strength of this study lies in its prospective design, which compared cytokine levels between lupus patients with LLDAS in CA and SACQ states, a comparison that has not been previously studied. This study utilized the throughput flow cytometry blood-based multiplex assay to analyze cytokines, which are key components in the pathogenesis of lupus. The selection of participants was rigorous, ensuring that all patients met the diagnostic criteria for LLDAS and that minimal confounding factors affected the analysis and study outcomes, as determined by strict exclusion criteria. Additionally, all patients were followed up for 6 months after blood collection to assess clinical symptoms and to ensure a comprehensive evaluation of all research participants.

This study has certain limitations. The relatively small sample size and short follow-up duration (6 months) may limit the statistical power and generalizability of the results. No sample size calculation was performed to assess the predictors of disease relapse, which might have reduced the ability to detect other contributing factors. The absence of a healthy and active SLE control group limits the ability to compare cytokine levels. Age and inequity in immunosuppressive drug use are other potential confounders; however, the ANCOVA was performed to minimize and identify the impact of immunosuppressive drugs on cytokine levels. The omission of the PGA is a limitation of this study, as it deviates from the classical SELENA-SLEDAI Flare Index (cSFI) [22,23]. In our cohort, different physicians performed enrollment and follow-up assessments, resulting in incomplete PGA data. Current recommendations emphasize that PGA scoring should be performed consistently, preferably by the same experienced physician, to ensure reliability [24]. To maintain methodological consistency and minimize missing data, PGA was therefore excluded from the SFI. Given its subjective and physician-dependent nature, and its limited ability to distinguish acute flares from persistent low-grade activity, we believe that the remaining SFI components were sufficient to capture clinically meaningful flares.

## 4. Materials and Methods

### 4.1. Study Design, Participants, and Eligibility Criteria

This prospective cohort study was conducted at the Rheumatology Clinic of Songklanagarind Hospital, Thailand, from January to December 2023. Inclusion criteria comprised adult patients with SLE aged ≥18 years who achieved LLDAS, defined as SLEDAI-2K ≤4, prednisolone dose ≤7.5 mg/day, stable immunosuppressive therapy, and PGA ≤1. LLDAS had to be sustained for at least 6 months. The exclusion criteria were (1) patients with overlapping syndromes, (2) active infections (e.g., tuberculosis and HIV), (3) pregnancy, and (4) end-stage renal disease requiring dialysis. Patients who had prior treatment with intravenous immunoglobulin, therapeutic plasma exchange, anti-B-cell therapy, or biologic therapy were also excluded due to the small number of patients. Patients were divided into two groups: CA—patients with a systemic lupus erythematosus disease activity index 2000 (SLEDAI-2K) score of 1–4 based on clinical symptoms only, and SACQ—patients who had a SLEDAI-2K score of 2 or 4 owing to the presence of anti-dsDNA antibodies and/or low complement levels, but without any clinical symptoms. The study flow diagram is shown in Figure S1. All the participants provided written informed consent. The Human Research Ethics Committee approved this study (approval no. REC 66-116-14-3), and this study was conducted in accordance with the principles of Good Clinical Practice.

### 4.2. Serology and Cytokine Measurement

The sera of SLE patients were collected for serology on the date of blood collection for cytokine measurement, including complement (C)-3 (0.9 to 1.8 g/L), C4 (0.1 to 0.4 g/L) using nephelometry (Atellica^®^ NEPH 630 System and BN System, Siemens Healthineers, Erlangen, Germany) and anti-dsDNA (Enzyme-Linked Immunosorbent Assay, upper limit of normal range < 100 IU/mL, (EUROIMMUN Medizinische Labordiagnostika AG, Lübeck, Germany). Clotted blood samples were centrifuged to obtain serum and stored at −80 °C within 6 h of blood collection; after completed enrolment, the serum was shipped for cytokine analysis including interleukin (IL)-1β, interferon (IFN)-α, IFN-γ, tumor necrosis factor (TNF)-α, monocyte chemotactic protein (MCP)-1 (CCL-2), IL-6, IL-8 (CXCL-8), IL-10, IL-12p70, IL-17A, IL-18, IL-23, and IL-33. The stored sera were thawed for the first time and immediately stained with antibodies according to the manufacturer’s protocol. Cytokine concentrations were quantified using a bead-based multiplex flow cytometry assay (Human Th Cytokine Panel, #740809, LEGENDplex™, BioLegend, San Diego, CA, USA). Flow cytometry acquisition was performed using a BD FACSVerse™ (Becton Dickinson Biosciences, San Jose, CA, USA). Raw flow cytometry data files (FCS 2.0 and 3.0) were analyzed using LEGENDplex™ (version 8.0) Data Analysis Software (BioLegend, San Diego, CA, USA). Cytokine concentrations were reported in picograms per milliliter (pg/mL), with a limit of quantification (LOQ) ranging from 0.01 to 30,000 pg/mL. Normal cytokine reference ranges were based on the manufacturer’s calibration dataset derived from a demographically matched healthy population and are shown in Table S6.

### 4.3. Weighted Gene Co-Expression Network Analysis

We applied Weighted Gene Co-expression Network Analysis (WGCNA) to examine the correlation patterns among 13 cytokines across 50 samples. Briefly, a pairwise correlation network was constructed using an optimal soft-thresholding power based on the scale-free topology criterion. Modules were identified from the Topological Overlap Matrix (TOM) through hierarchical clustering and dynamic tree cutting. The relationships between module and clinically interesting traits were evaluated using corPvalueStudent, yielding a protein significance (PS) score and *p*-value. The module–trait correlation and the PS score for each intertest clinical trait were re-analyzed using a multiple-comparison correction method based on the Benjamin–Hochberg procedure. Statistical significance was defined as False Discovery Rate (FDR) < 0.05.

### 4.4. Outcome Measurement

The primary objective of this study was to compare cytokine levels in SLE patients across the LLDAS subgroups (CA and SACQ). The secondary objective was to identify clinical factors and cytokine levels that predicted a 6-month disease flare based on cytokine measurements. All patients underwent standard care protocols for at least 6 months. The censored date was identified as 6 months after cytokine measurement or a disease flare-up. Patients were categorized as sustained LLDAS if maintaining LLDAS for ≥50% of total follow-up time, whereas non-sustained LLDAS was defined as LLDAS <50%. Disease flare was defined according to the Safety of Estrogens in Lupus Erythematosus National Assessment (SELENA) flare index (SFI) by an increase of ≥3 points in the SLEDAI without physician global assessment (PGA) measurement, if a total SLEDAI score ≥ 12, indicating a severe flare. If a new flare occurred in any organ not captured by the SLEDAI, the attending physician’s judgment, supported by an escalation of glucocorticoids or immunosuppressive therapy, was also defined as a disease flare-up.

### 4.5. Statistical Analyses

Categorical variables were compared using Pearson’s chi-squared test or Fisher’s exact test and are presented as counts and percentages. Normality of continuous variables was assessed using the Shapiro–Wilk test. Continuous variables were compared using Student’s *t*-test (Welch correction) or the Wilcoxon rank-sum test, as appropriate. Results are presented as mean (standard deviation [SD]) or median (interquartile range [IQR]), depending on the data distribution. The correlation between cytokines was calculated using Spearman’s rank correlation coefficient. To identify candidate biomarkers that predict disease flare-ups, we selected cytokines with significant PS from WGCNA for further analysis using the Area Under the ROC Curve (AUC) and the Youden index to determine the optimal cutoff. To assess potential confounding effects of clinical variables on cytokine levels, analysis of covariance (ANCOVA) was performed. Univariable and multivariable analyses were performed using Cox regression with backward elimination, and the results are expressed as Hazard Ratios (HRs). Statistical significance was defined as *p* < 0.05. We used the R package version 4.3.1. software (R Foundation for Statistical Computing, Vienna, Austria) for analysis.

## 5. Conclusions

Patients with SLE exhibit complex immune system abnormalities. Even when LLDAS is the current treatment goal, patients with LLDAS with clinical symptoms were found to have higher cytokine levels than those without symptoms, and are also at a higher risk of disease relapse. Predictive factors for disease relapse included non-sustained LLDAS or IL-6 levels ≥ 45.1 pg/mL. Therefore, using clinical characteristics, such as disease remission duration, along with IL-6 level assessment to evaluate the risk of disease relapse in patients with LLDAS could improve the management of patients with lupus. However, further studies with larger populations are required to validate the accuracy of these findings.

## Figures and Tables

**Table 1:** Time to LLDAS, clinical symptoms, and laboratory test results on the cytokine level test date.

Characteristics	LLDAS	CA	SACQ	*p*-Value
	*n* = 50 (%)	*n* = 25 (%)	*n* = 25 (%)	
Median (IQR) LLDAS achieved duration, y	3.1 (1.5, 6.7)	2.5 (0.6, 3.4)	4.6 (3.1, 7.3)	0.02 ^a^
Non-sustained LLDAS	22 (44.0)	12 (48.0)	10 (40.0)	0.57
Median (IQR) SELENA-PGA	0.5 (0.5, 1)	1 (0.5, 1)	0.5 (0, 1)	0.01 ^a^
Median (IQR) SLEDAI-2K	3.5 (2, 4)	4 (2, 4)	2 (2, 4)	0.34
Median (IQR) clinical SLEDAI-2K	0.5 (0, 2)	2 (1, 2)	0 (0, 0)	
Serology status at LLDAS state				
Anti-dsDNA +ve alone	10 (20.0)	6 (24.0)	4 (16.0)	0.48
Low complement levels alone	18 (36.0)	7 (28.0)	11 (44.0)	0.24
Anti-dsDNA +ve and low complement	10 (20.0)	0 (0.0)	10 (40.0)	
Serologically inactive	12 (24.0)	12 (48.0)	0 (0.0)	
Organ involvement, blood collection for cytokines analysis date				
Mucocutaneous	1 (2.0)	1 (4.0)	0 (0.0)	
Alopecia	7 (14.0)	7 (28.0)	0 (0.0)	
Rash	5 (10.0)	5 (20.0)	0 (0.0)	
Musculoskeletal	4 (8.0)	4 (16.0)	0 (0.0)	
Pleurisy	1 (2.0)	1 (4.0)	0 (0.0)	
Thrombocytopenia	2 (4.0)	2 (8.0)	0 (0.0)	
Leukopenia	6 (12.0)	6 (24.0)	0 (0.0)	
Treatment during LLDAS				
Prednisolone	45 (90.0)	22 (88.0)	23 (92.0)	1
Median (IQR) prednisolone, mg	5 (5, 7.5)	5 (5, 7.5)	5 (5, 7.5)	0.3
Azathioprine	23 (46.0)	7 (28.0)	16 (64.0)	0.02 ^a^
Mycophenolate mofetil	5 (10.0)	3 (12.0)	2 (8.0)	1
Methotrexate	2 (4.0)	2 (8.0)	0 (0.0)	0.5
Hydroxychloroquine	45 (90.0)	22 (88.0)	23 (92.0)	1
Mean (SD) time to censored, wk	22.4 (6.3)	23.1 (4.9)	21.7 (7.5)	0.47

IQR, interquartile range; LLDAS, Lupus Low Disease Activity State; CA, Clinically Active; SACQ, Serologically Active Clinically Quiescent; SELENA-PGA, Safety of Estrogens in Lupus Erythematosus National Assessment—Physician’s Global Assessment; wk, week; SD, standard deviation. ^a^ *p* < 0.05.

**Table 2:** Baseline characteristics of patients with LLDAS between the flare-up and clinically stable groups.

Clinical Characteristics	LLDAS	Disease-flare	Inactive	*p*-Value
	*n* = 50 (%)	*n* = 9 (%)	*n* = 41 (%)	
Age at diagnosis, y	30.5 (10.7)	28.5 (11.0)	31 (10.7)	0.54
SLEDAI at diagnosis	10 (7, 14.5)	8 (7, 15)	10 (7, 13)	0.57
Group of LLDAS				0.46
CA	25 (50.0)	6 (66.7)	19 (46.3)	
SACQ	25 (50.0)	3 (33.3)	22 (53.7)	
Median (IQR) Physician global assessment	0.5 (0.5, 1)	1 (0.5, 1)	0.5 (0.5, 1)	0.15
LLDAS duration (y)	3.1 (1.5, 6.7)	1.9 (0.8, 3)	3.4 (2, 7.3)	0.04 ^a^
LLDAS-to-total follow-up ratio	0.5 (0.3)	0.3 (0.3)	0.6 (0.3)	0.02 ^a^
Non-sustained LLDAS	22 (44.0)	7 (77.8)	15 (36.6)	0.03 ^a^
Serology status and treatment at enrolment				
Anti-dsDNA positive	10 (20.0)	1 (11.1)	9 (22.0)	0.66
Low complement level	18 (36.0)	5 (55.6)	13 (31.7)	0.25
Corticosteroids	15 (30.0)	3 (33.3)	12 (29.3)	0.84
Azathioprine	23 (46.0)	5 (55.6)	18 (43.9)	0.71
Hydroxychloroquine	45 (90.0)	9 (100.0)	36 (87.8)	0.57
Cytokines pg/mL				
IL-1β	21.3 (9.9, 42.6)	33.4 (10.7, 77.8)	21.3 (9.6, 39.6)	0.69
IFN-α	17.6 (9, 33.3)	15.4 (14.2, 39.8)	18.0 (8.4, 32.8)	0.53
IFN-γ	10.2 (5.6, 34.2)	9.4 (3.6, 97.3)	10.7 (6.6, 33.7)	0.82
TNF-α	8.3 (3.6, 40.1)	8.3 (3.9, 47.3)	8.3 (3.5, 38.2)	0.92
MCP-1	2468.2 (1384.3)	2258.1 (785.5)	2514.4 (1487.2)	0.62
IL-6	43.8 (23.6, 78.5)	59.0 (45.1, 130.6)	39.9 (23.2, 73.9)	0.18
IL-8	1399 (607.7, 2408.1)	955.5 (717.7, 1661.6)	1479.8 (515.3, 2880.1)	0.54
IL-10	28.1 (13.2, 74.9)	19.8 (10.8, 104.7)	29.8 (13.3, 66.8)	0.9
IL-12p70	18.2 (7.4, 39.6)	18.0 (7.5, 55.1)	18.5 (7.4, 37.8)	0.99
IL-17A	5.0 (1.3, 8.4)	5.6 (2.4, 10.3)	4.4 (1.2, 8.1)	0.69
IL-18	1104.4 (455.2)	1076.6 (344.9)	1110.5 (479.4)	0.84
IL-23	33.8 (21.9, 50.9)	35.7 (12.7, 81.7)	32.6 (23.7, 46.8)	1
IL-33	501.4 (220.1, 964.2)	725.9 (220.3, 1569.3)	495.4 (220.1, 844.8)	0.45

CA, clinically active; IFN, interferon; IL, interleukin; IQR, interquartile range; LLDAS, lupus low disease activity state; MCP, monocyte chemotactic protein; SACQ, serologically active clinically quiescent; SLEDAI, Systemic Lupus Erythematosus Disease Activity; TNF, tumor necrosis factor. ^a^ *p* < 0.05.

**Table 3:** Univariable and multivariable analyses to identify factors associated with disease flare-ups, along with HR calculation.

Variables	Univariate HR (95–CI)	Univariable *p*-Value	Multivariate HR (95–CI)	Multivariable *p*-Value
CA vs. SACQ	1.6 (0.41–6.6)	0.490	Not included	
Physician global assessment	1.1 (0.59–2.20)	0.690	Not included	
Non-sustained LLDAS	5.0 (1.00–24.00)	0.044	12.38 (2.22–69.10)	0.004 ^a^
IFN-γ ≥ 97.3 pg/mL	4.8 (1.2–19.0)	0.028	Remove by backward elimination.	
IL-6 ≥ 45.1 pg/mL	4.4 (0.9–21.0)	0.070	11.47 (1.99–66.29)	0.006 ^a^
IL-10 ≥ 131.1 pg/mL	13.0 (2.4–74.0)	0.003	Remove by backward elimination.	
IL-23 ≥ 81.7 pg/mL	3.4 (0.8–13.0)	0.080	Remove by backward elimination.	

CA, clinically active; CI, confidence interval; HR, hazard ratio; IFN, interferon; IL, interleukin; LLDAS, lupus low disease activity state; SACQ, serologically active clinically quiescent. ^a^ *p* < 0.05.

## Data Availability

The data that support the findings of this study are available from the corresponding author upon reasonable request.

## Supplementary Materials

The following supporting information can be downloaded at: https://www.mdpi.com/article/10.3390/ijms27093913/s1.
