# Supplementary material for: Distinct Cytokine Profiles in Lupus Low Disease Activity State Subgroups Identify Patients at Risk for Disease Flare
Source: Int J Mol Sci. 2026 Apr 28;27(9):3913. doi: 10.3390/ijms27093913 (PMC13164232; doi:10.3390/ijms27093913)
Supplement: Supplementary file 1 [file ijms-27-03913-s001.zip › ijms-4204633-supplementary.pdf]

Supplementary material

Supplementary Table S1: Comparison of cytokine levels between the CA and SACQ groups.

Cytokines (pg/mL)	LLDAS (n = 50)	CA (n = 25)	SACQ (n = 25)	method	p-value	FDR
IL-1B	21.3 (9.9,42.6)	29.6 (16.2,63.1)	16.3 (8.9,31.4)	Wilcoxon rank-sum	0.055	0.177
IFN-α	17.6 (9,33.3)	18 (9.1,46.4)	16.5 (8.3,29.7)	Wilcoxon rank-sum	0.286	0.417
IFN-γ	10.2 (5.6,34.2)	14.7 (5.3,62.4)	9.1 (6.3,32)	Wilcoxon rank-sum	0.6	0.653
TNF-α	8.3 (3.6,40.1)	9.7 (3.9,38.2)	8 (3.3,45.8)	Wilcoxon rank-sum	0.698	0.698
MCP-1	2190.9 (1638.4,3155.1)	2214.1 (1906.6,2814.2)	2165.4 (1301.1,3411.6)	Wilcoxon rank-sum	0.415	0.543
IL-6	43.8 (23.6,78.5)	59.9 (32.9,91.5)	27.3 (21.3,58.6)	Wilcoxon rank-sum	0.035a	0.177
IL-8	1399 (607.7,2408.1)	2179.7 (818.1,3823.4)	1190.5 (476.6,1610.8)	Wilcoxon rank-sum	0.049a	0.177
IL-10	28.1 (13.2,74.9)	46.1 (10.8,105.4)	26.1 (13.3,41.5)	Wilcoxon rank-sum	0.187	0.410
IL-12p70	18.2 (7.4,39.6)	28.7 (5.1,63.3)	13.4 (7.6,25.2)	Wilcoxon rank-sum	0.148	0.385
IL-17A	5 (1.3,8.4)	5.9 (3.3,9.6)	2.4 (0.9,6.8)	Wilcoxon rank-sum	0.051	0.177
IL-18	1070.1 (773.4,1361.6)	973.9 (682.1,1330.5)	1182 (891.1,1372.9)	Welch t-test	0.309	0.417
IL-23	33.8 (21.9,50.9)	39.8 (17.2,72)	30.4 (26.7,37.5)	Wilcoxon rank-sum	0.535	0.636
IL-33	501.4 (220.1,964.2)	642.8 (196,1631.2)	418.4 (220.3,830.1)	Wilcoxon rank-sum	0.24	0.417

CA, clinically active; IFN, interferon; IL, interleukin; LLDAS, lupus low disease activity state; SACQ, serologically active clinically quiescent; MCP, monocyte chemotactic protein; TNF, tumor necrosis factor

[a]$p < 0.05$

Supplementary Table S2: The protein significant (PS) score between cytokines and the clinical trait of interest.

Proteins	Module	PS.CA	FDR.PS.CA	PS.SACQ	FDR.PS.SACQ	PS.DORIS	FDR.PS.DORIS
TNFA	blue	-0.14	0.344	0.04	0.770	0.05	0.750
IL6	blue	0.14	0.328	0.14	0.333	-0.11	0.441
IL1B	blue	0.20	0.158	-0.35	0.012	-0.19	0.191
MCP1	brown	0.10	0.498	0.11	0.436	0.02	0.873
IL8	brown	0.27	0.055	-0.08	0.604	-0.17	0.226
IFNG	turquoise	0.24	0.097	0.04	0.797	-0.21	0.142
IFNA	turquoise	0.24	0.092	0.07	0.610	-0.24	0.097
IL17A	turquoise	-0.08	0.577	-0.14	0.324	-0.14	0.331
IL10	turquoise	0.29	0.045	0.01	0.957	-0.16	0.281
IL12p70	turquoise	0.07	0.620	0.07	0.609	-0.15	0.314
IL18	turquoise	-0.03	0.856	-0.12	0.420	-0.01	0.967
IL23	turquoise	0.21	0.138	-0.07	0.654	-0.18	0.215
IL33	turquoise	0.16	0.277	-0.06	0.665	-0.16	0.254

Proteins	Module	PS.Flare	FDR.PS.Flare	PS.Attainment	FDR.PS.Attainment	PS.PGA	FDR.PS.PGA
TNFA	blue	-0.12	0.423	-0.25	0.085	0.01	0.947
IL6	blue	-0.07	0.627	-0.23	0.109	0.09	0.531
IL1B	blue	0.01	0.965	-0.32	0.023	0.14	0.321
MCP1	brown	-0.09	0.550	-0.02	0.902	-0.03	0.811
IL8	brown	-0.05	0.715	-0.23	0.110	-0.07	0.640
IFNG	turquoise	0.10	0.470	-0.08	0.593	0.32	0.024
IFNA	turquoise	0.06	0.685	-0.26	0.074	0.52	0.000
IL17A	turquoise	0.05	0.729	-0.05	0.713	-0.05	0.726
IL10	turquoise	0.04	0.799	-0.15	0.289	0.69	<0.001

IL12p70	turquoise	0.03	0.862	-0.10	0.510	0.45	0.001		
IL18	turquoise	-0.01	0.937	0.08	0.593	0.02	0.868		
IL23	turquoise	0.00	0.984	-0.11	0.458	0.64	<0.001		
IL33	turquoise	0.00	0.994	-0.21	0.144	0.51	<0.001		

**Proteins**	**Module**	**PS. Anti-DNA**	**FDR.PS. Anti-DNA**	**PS. Complement**	**FDR.PS. Complement**	**PS. SLEDAI**	**FDR.PS. SLEDAI**	**PS. CSLEDAI**	**FDR.PS. CSLEDAI**
TNFA	blue	0.01	0.940	0.10	0.496	-0.25	0.085	-0.16	0.273
IL6	blue	-0.08	0.585	-0.11	0.445	-0.11	0.441	0.11	0.449
IL1B	blue	0.05	0.734	-0.18	0.223	-0.06	0.681	0.15	0.306
MCP1	brown	-0.13	0.352	-0.04	0.806	0.05	0.708	0.22	0.128
IL8	brown	0.15	0.314	-0.25	0.077	0.06	0.703	0.21	0.148
IFNG	turquoise	0.15	0.290	-0.19	0.175	0.07	0.612	0.22	0.130
IFNA	turquoise	0.05	0.731	-0.03	0.825	0.02	0.891	0.14	0.316
IL17A	turquoise	-0.07	0.653	-0.09	0.513	0.12	0.415	-0.04	0.805
IL10	turquoise	0.03	0.837	0.09	0.516	0.04	0.801	0.16	0.276
IL12p70	turquoise	0.00	0.977	0.00	0.997	0.10	0.503	-0.02	0.905
IL18	turquoise	-0.39	0.005	-0.07	0.642	0.14	0.336	0.18	0.223
IL23	turquoise	0.04	0.807	0.03	0.828	0.00	0.977	0.08	0.586
IL33	turquoise	0.06	0.684	-0.02	0.907	0.01	0.969	0.03	0.848

**Proteins**	**module**	**PS.Steroid**	**FDR.PS.Steroid**	**PS. AZA**	**FDR.PS.AZA**	**PS.MMF**	**FDR.PS.MMF**	**PS.HCQ**	**FDR.PS.HCQ**
TNFA	blue	0.06	0.673	0.00	0.987	0.07	0.649	0.17	0.247
IL6	blue	0.02	0.878	-0.09	0.517	0.40	0.004	0.05	0.735
IL1B	blue	0.04	0.779	-0.05	0.740	0.31	0.028	0.05	0.719
MCP1	brown	-0.25	0.081	0.03	0.826	-0.18	0.200	-0.10	0.509

IL8	brown	-0.17	0.246	-0.26	0.068	0.24	0.093	-0.26	0.071
IFNG	turquoise	0.08	0.598	0.05	0.730	-0.04	0.757	0.07	0.607
IFNA	turquoise	0.18	0.213	-0.02	0.903	0.07	0.647	0.01	0.956
IL17A	turquoise	0.19	0.189	0.16	0.264	0.00	0.989	0.09	0.542
IL10	turquoise	0.07	0.625	-0.12	0.387	0.07	0.650	0.06	0.682
IL12p70	turquoise	0.20	0.164	0.01	0.959	0.03	0.843	0.08	0.592
IL18	turquoise	-0.15	0.283	-0.08	0.578	0.05	0.719	-0.02	0.876
IL23	turquoise	0.08	0.591	-0.18	0.203	0.10	0.489	0.06	0.689
IL33	turquoise	0.18	0.203	-0.01	0.928	0.13	0.362	0.01	0.944

AZA, azathioprine; CA, clinically active; DORIS, Definitions of Remission in systemic lupus erythematosus; HCQ, hydroxychloroquine; IFN, interferon; IL, interleukin; LLDAS, lupus low disease activity state; MCP, monocyte chemotactic protein; MMF, mycophenolate mofetil; PGA, physician global assessment; PS, protein score; SACQ, serologically active clinically quiescent; SLEDAI, systemic lupus erythematosus disease activity index 2000 ; TNF, tumor necrotic factor

Supplementary Table S3: Clinical characteristics and serological findings in nine patients who experienced 6-month disease flare-ups.

Characteristics	1	2	3	4	5	6	7	8	9
Group	CA	SACQ	SACQ	CA	CA	SACQ	CA	CA	CA
LLDAS/total follow-up ratio	0.2	0.97	0.05	0.2	0.36	0.2	0.04	0.19	0.84
Sustaine LLDAS	no	yes	no	No	no	no	no	no	yes
Time to flare (wk)	14	13	13	20	14	22	18	13	24
Organ involvement	Skin	LN	LN, skin	Skin	Skin	GI	LN	Skin	Joint/skin
SLEDAI-2K score	6	6	16	7	7	10	10	8	6
SFI	Mild	Severe	Severe	Mild	Mild	Severe	Moderate	Mild	Mild
Complement level	Low	Low	Low	Low	Low	Low	Normal	Normal	Normal
Anti-dsDNA	Normal	Normal	Normal	Positive	Normal	Normal	Normal	Normal	Normal
IL-6 levels (pg/mL)	21.11	27.345	141.813	130.641	194.84	52.112	75.72	59.005	45.117

CA, clinically active, GI, gastrointestinal; LN, lymph nodes; LLDAS, lupus low disease activity state; SACQ, serologically active clinically quiescent; SFI, Safety of Estrogens in Lupus Erythematosus National Assessment/Systemic Lupus Erythematosus Disease Activity Index; SLEDAI-2K, systemic lupus erythematosus disease activity index 2000; wk, week; IL, interleukin

Supplementary Table S4: AUC, sensitivity, specificity, PPV, and NPV of the cytokine cutoff level for predicting 6-month disease flare-up.

Cytokines	Cutoff levels	AUC	Sensitivity	Specificity	PPV	NPV
IL-1β	33.4 pg/mL	0.545	0.56	0.68	0.28	0.88
IFN-α	9.6 pg/mL	0.569	0.89	0.34	0.23	0.93
IFN-γ	97.3 pg/mL	0.474	0.33	0.95	0.60	0.86
TNF-α	32.6 pg/mL	0.512	0.44	0.73	0.27	0.86
MCP-1	1467.9 pg/mL	0.458	1.00	0.22	0.22	1.00
IL-6	45.1 pg/mL	0.645	0.78	0.56	0.28	0.92
IL-8	471.8 pg/mL	0.434	1.00	0.20	0.21	1.00
IL-10	131.2 pg/mL	0.515	0.22	1.00	1.00	0.85
IL-12p70	55.1 pg/mL	0.503	0.33	0.85	0.33	0.85
IL-17A	10.3 pg/mL	0.545	0.33	0.85	0.33	0.85
IL-18	1053.6 pg/mL	0.509	0.67	0.51	0.23	0.87
IL-23	81.7 pg/mL	0.501	0.33	0.93	0.50	0.86
IL-33	752.9 pg/mL	0.583	0.56	0.68	0.28	0.88

AUC, area under the curve; IFN, interferon; IL, interleukin; MCP, monocyte chemotactic protein; NPV, negative predictive value; PPV, positive predictive value; TNF, tumor necrosis factor

Supplementary Table S5: The normal cytokine reference ranges were based on the manufacturer's calibration dataset derived from a demographically matched healthy population.

Cytokines	Normal (Range)
IL-1β	1.59-13.88
IFN-α	1.88-7.60
IFN-γ	1.30-17.09
TNF-α	2.47-22.28
MCP-1	40.73-318.12
IL-6	2.19-12.88
IL-8	2.00-80.71
IL-10	1.96-28.48
IL-12p70	0.79-8.70
IL-17A	0.81-16.84
IL-18	50.78-166.58
IL-23	4.55-16.30
IL-33	9.65-104.96

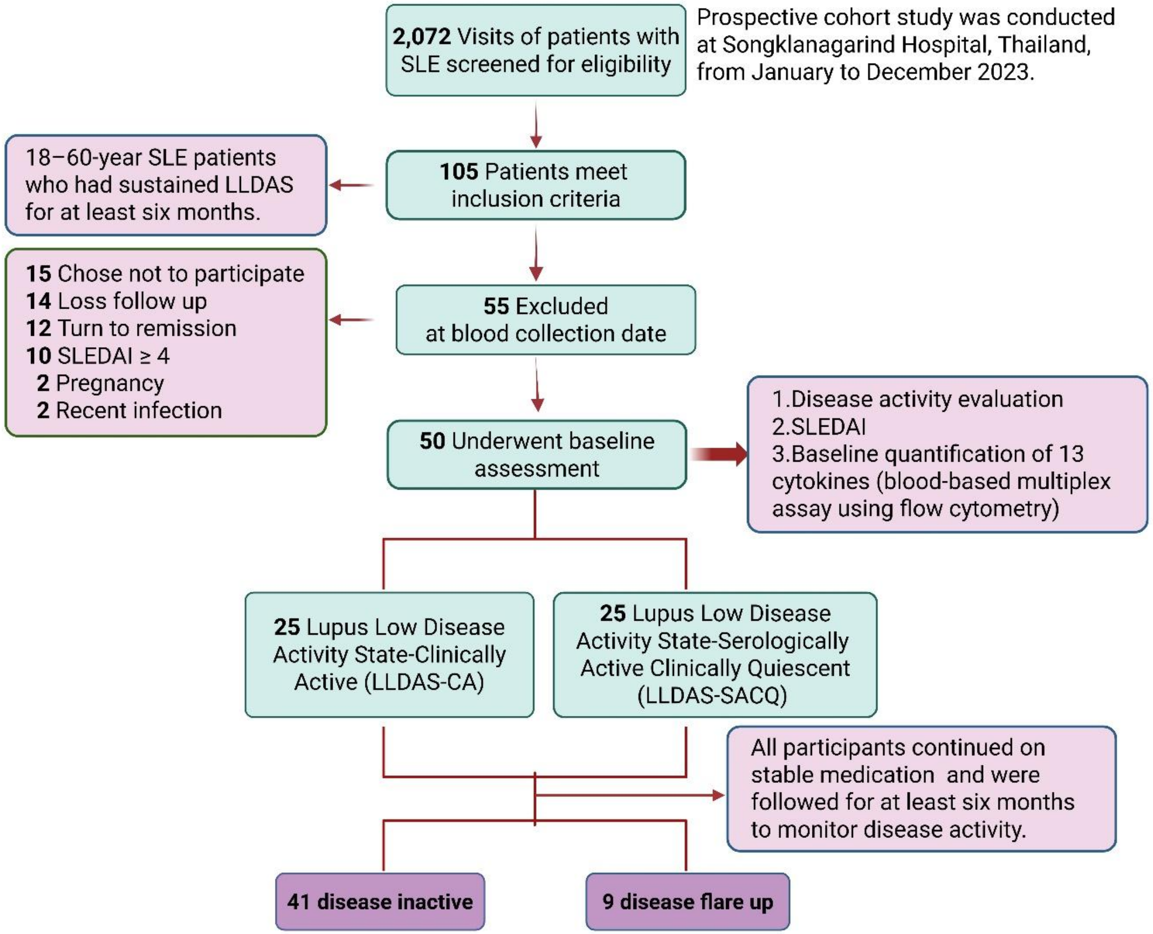

Supplementary Figure S1: A flow diagram of the study participants. CA, clinically active; LLDAS, lupus low disease activity state; SACQ, serologically active clinically quiescent; SLE, Systemic Lupus Erythematosus; SLEDAI, The Systemic Lupus Erythematosus Disease Activity Index score.

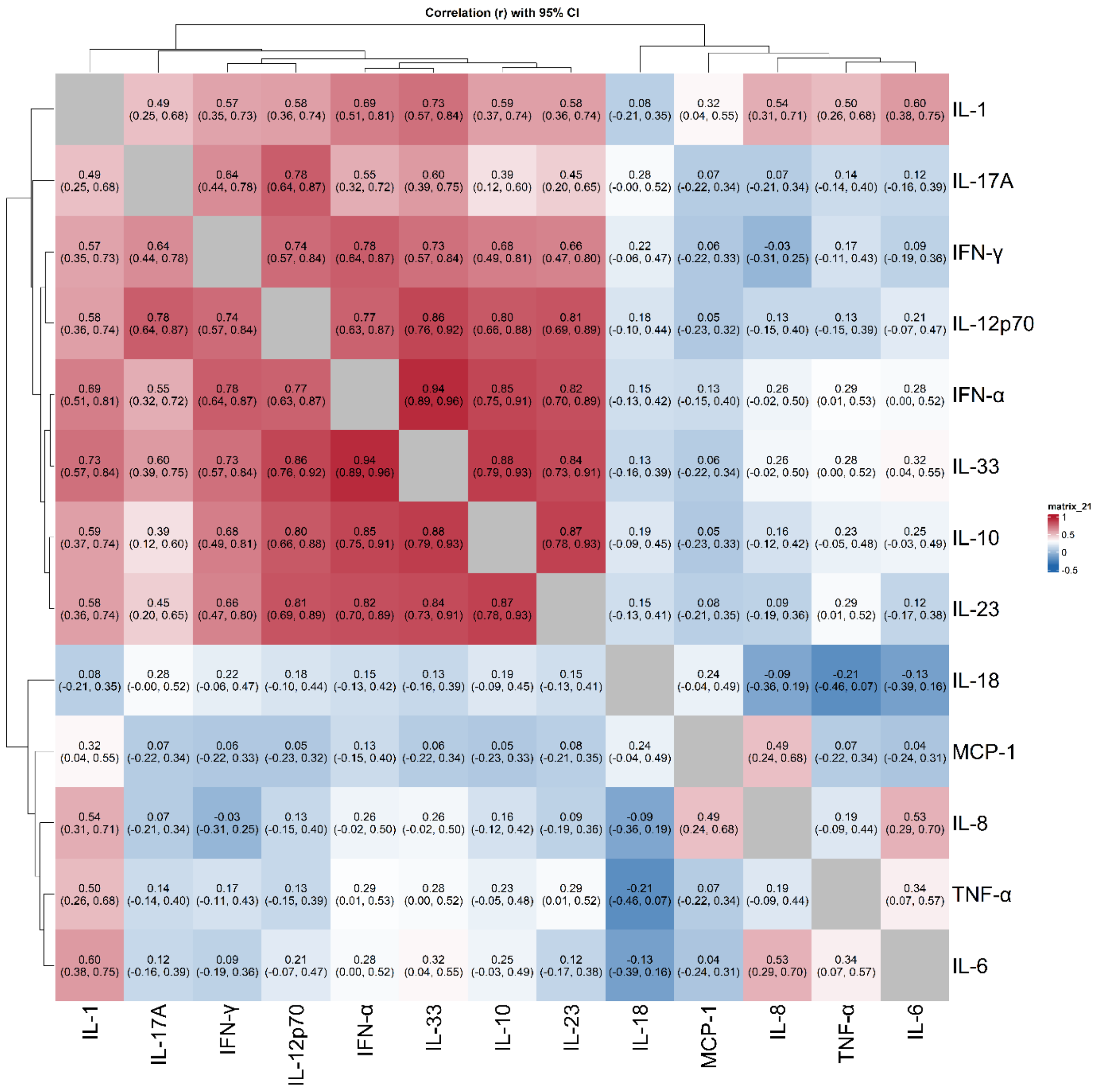

Supplementary Figure S2: Spearman's rank correlation coefficient shows the correlation between cytokine–cytokine interactions. IFN, interferon; IL, interleukin; MCP, monocyte chemotactic protein; TNF, tumor necrosis factor.
